# Serological cross-reactivity between Crimean-Congo haemorrhagic fever virus and Nairobi sheep disease virus glycoprotein C

**DOI:** 10.3389/fimmu.2024.1423474

**Published:** 2025-01-20

**Authors:** Emmanuel A. Maze, George Booth, Georgina Limon, Sandra Belij-Rammerstorfer, Simona R. Tchakarova, Tsviatko Alexandrov, Clare Browning, Ginette Wilsden, Anna B. Ludi, Bryan Charleston, Teresa Lambe

**Affiliations:** ^1^ The Pirbright Institute, Woking, United Kingdom; ^2^ Oxford Vaccine Group, Department of Paediatrics, University of Oxford, Oxford, United Kingdom; ^3^ Bulgarian Food Safety Agency, Sofia, Bulgaria; ^4^ Pandemic Sciences Institute, Oxford, United Kingdom

**Keywords:** Crimean-Congo haemorrhagic fever, CCHF, Nairobi Sheep disease, cross-reaction, antibody response, *Orthonairovirus*

## Abstract

**Introduction:**

Crimean-Congo haemorrhagic fever virus (CCHFV) and Nairobi sheep disease virus (NSDV) are orthonairoviruses of concern, able to cause haemorragic fever disease in humans and sheep, respectively. CCHFV and NSDV cocirculating in small ruminant populations across South Asia and East Africa. Cross-reactivity to viruses of the Orthonairovirus genus can potentially interfere with serological assays when employed for serosurveillance in regions where two or more genus members overlap in their distribution.

**Methods:**

In this study, sheep sera sampled from a region of confirmed CCHFV circulation and NSDV absence were utilized, thereby eliminating the possibility of co-exposure. Field sera were tested against in-house anti-NSDV ELISAs specific to the nucleoprotein (NSDV NP) and glycoprotein C (NSDV Gc) antigens as well as an in-house NSDV 80% plaque reduction neutralization test (PRNT_80_). We assessed whether there is a correlation between CCHFV- and NSDV-specific ELISAs. Furthermore, epitopes-derived from CCHFV antigens for sheep antibody that were available from the literature were analyzed.

**Results:**

When comparing NSDV antigen-specific antibody responses against previously tested CCHFV antigen-specific antibody responses, a strong positive correlation was observed between the Gc-specific responses, while a weak positive correlation was observed between the NP-specific responses. Consequently, NP-specific ELISAs have a higher assay specificity compared to Gc-specific ELISAs, making them more suitable for serosurveillance in regions where multiple orthonairoviruses co-circulate. Crucially, only one seropositive sample to NSDV Gc-specific out of a set of 224 (0.4%) showed a neutralizing capacity at the lowest serum dilution (1:8), suggesting these field sera have not been exposed to NSDV. Based on an analysis of known epitopes in NP targeted by antibodies in sheep serum, we propose that NP is less cross-reactive because dominant epitopes are highly dissimilar between CCHFV and NSDV.

**Discussion:**

Gc exhibited a strong cross-reaction while the NP was weakly cross-reactive due to dominant epitopes being highly dissimilar between CCHFV and NSDV. Our in-house PRNT80 assay can could be used as a confirmatory test in regions where CCHFV and NSDV circulate.

## Introduction

The *Nairoviridae* family represents a group of tick-borne arboviruses expected to expand in their distribution with the effect of global warming. These viruses display a structure made of a negative-sense single-stranded tri-segmented RNA genome encapsulated in a lipid-based envelope (1). Of this family, recently classified into the *Hareavirales* order of the class *Bunyaviricetes* (2), the *Orthonairovirus* genus contains fifteen distinct viral species (3), two of which are of most concern for human and animal health: Crimean-Congo haemorrhagic fever virus (CCHFV) and Nairobi sheep disease virus (NSDV).

CCHFV is the most notable orthonairovirus, able to cause Crimean-Congo haemorrhagic fever (CCHF) in humans. Fever, nausea and myalgia occur in the early-stage of human CCHF infections, before the disease progresses with haemorrhagic symptoms (petechiae, hematomas), resulting in a mortality rate of 3-30% (4). Birds, ruminants and other small mammals have been shown to support CCHFV infections without the onset of symptoms, facilitating a tick-vertebrate-tick life cycle through which CCHFV circulates (5). CCHFV has a reported widespread distribution in small ruminant populations across Eastern Europe, Southern Asia and the African continent, following the distribution of its principal vectors, the *Hyalomma* species of ticks (6), and more recently, *Rhipicephalus* species (7).

The second orthonairovirus of concern, NSDV, is the aetiological agent of Nairobi sheep disease (NSD). Several stages characterize an NSD infection, where small ruminants display fever, lethargy and depressive behavior before severe haemorrhagic gastroenteritis ensues (8). While small ruminants in enzootic regions are often protected by maternal antibodies, NSD is highly lethal in naïve individuals, with mortality rates that can reach 90% as previously recorded within populations imported into enzootic areas (8, 9). Historical evidence suggests that symptomatic NSDV infections in humans are extremely rare, with exposed laboratory personnel working on NSDV in the 1970s being one of the few previously reported cases, where NSDV was determined as the cause of a febrile illness (10). NSDV has an understudied tick-vertebrate-tick life cycle, in which the principal tick vectors *Rhipicephalus* and *Haemaphysalis* species, localize its distribution in ruminant populations to East Africa and South Asia (where the virus is known as the Ganjam virus), respectively (11).

Few serological assays have been developed to allow the analysis of antibody dynamics over the course of infections as well as the detection of previous exposures to bunyaviruses including Rift valley fever virus (RVFV), CCHFV, Cache valley virus, Akabane virus, Schmallenberg virus and NSDV (12). Of note, NSDV-specific antibodies remain detectable in sheep for over a year post infection (13); hence testing small ruminants offers a possible method to survey NSDV circulation. The format of such serological assays includes virus neutralization tests, indirect immunofluorescence assays and Enzyme Linked Immunosorbent Assays (ELISAs) using virus preparation or nucleoprotein (NP) as bait (12). Recently, work by Belij-Rammerstorfer et al. (14) has utilized the Glycoprotein C (Gc) as a test antigen in an in-house ELISA; developed as a sensitive and specific alternative to NP-based ELISAs for assessing CCHFV circulation in small ruminants.

A disadvantage of serological assays lies with cross-reactivity. It happens when serum antibodies react to antigens from a virus the animal has not been exposed to, which may cause false positive misclassification of tested animals. Such cross-reactivities have been reported for members of the *Orthonairovirus* genus in the late 1970s and the early 1980s by means of immunofluorescence, haemagglutination inhibition and virus neutralization tests, using a combination of mouse ascetic fluids raised against a few orthonairoviruses and their corresponding antigens (15, 16). More recently, cross-reactivity has been demonstrated experimentally between related orthonairoviruses with overlapping distributions, including CCHFV, NSDV and Dugbe virus (DUGV); with the latter circulating in ruminant populations in Africa including Uganda (17), Ghana (18), Kenya (19), South Africa (20) without eliciting a symptomatic infection in this host species (21, 22). In fact, Hartlaub et al. observed that serum antibodies from a sheep immunized against DUGV, binds to cells expressing CCHFV glycoprotein precursor (CCHFV GPC), by employing a commercial indirect immunofluorescent assay; and to CCHFV NP by western blot (21). Moreover, sera raised against NSDV and DUGV from experimentally infected and immunized ruminants displayed cross-neutralizations when assayed for their ability to neutralize each virus by 80% plaque reduction neutralization tests (PRNT_80_), hence reducing the analytical specificity of these assays and possibly causing false-positive misclassification (22). Advancing on such experimental findings, cross-reactivity has been investigated using bovine field sera from a region of CCHFV and DUGV co-circulation (23). While no correlation was identified between CCHFV- and DUGV-specific antibody responses for respective in-house NP-based ELISAs, the commercial CCHFV Gc-based immunofluorescence assay was highly cross-reactive. However, since CCHFV and DUGV co-circulate in the region from that study, the possibility of a co-infection could not be excluded.

To investigate the effect cross-reactivity has on commonly used field serological assays, we used the understudied CCHFV and NSDV cross-reaction as a model, focusing on a sample set from a region where CCHFV, but neither NSDV nor DUGV are circulating. In this set-up, any CCHFV seropositive animals that tested positive for NSDV would be the result of cross-reactivity. Since NSDV has never been reported in Europe, a large set of sheep sera from Bulgaria, collected as part of a serosurvey in 2017, and previously screened against CCHFV NP and Gc antigens (14), were tested against in-house NSDV NP-specific and Gc-specific IgG ELISAs. The possible cross-reaction was also tested on an in-house PRNT_80_ assay, allowing us to assess whether CCHFV-endemic field sera can cross-neutralize NSDV *in vitro*. In addition to this, a small subset of sera from sheep experimentally infected with NSDV were tested with CCHFV NP-specific and Gc-specific ELISAs, in this case, animal sera that react to CCHFV antigens would be the result of a cross-reaction. While a commercial CCHFV-specific serological test was employed as gold standard, we also utilized a formaldehyde-inactivated whole NSDV as antigen in ELISA to confirm seroconversion of NSDV-infected sheep. Finally, potential causes for the observed cross-reactivity were explored by means of protein deglycosylation and analysis of available sequences of epitopes targeted by serum antibodies.

## Materials and methods

### Viruses and cells

NSDV isolate ND66 PC9 was used; initially obtained from Dr Piet van Rijn, Central Veterinary Institute of Wageningen, Netherlands (24). NSDV was passaged three times on BHK21, with subsequent virus stocks used in both back-titration and neutralization tests. The virus was prepared as follows: BHK21 were seeded in 175cm^2^ flasks and cultured until 90-100% of confluence, then they were washed in PBS and 15 ml of virus diluted 1:1000 in FBS- and antibiotic-free Glasgow minimum essential medium (GMEM, Cat # G5154, Sigma-Aldrich) was added onto cells. The inoculum was removed after an hour and cells were cultured in 45 ml of complete GMEM containing 2% FBS. Cells were kept for 3 days until cytopathic effects (CPE) appeared. The medium containing virus particles was harvested and clarified by centrifuged at 3500 × g for 10 minutes to remove cellular debris.

BSR-T7 cells were cultured in complete DMEM made of Dulbecco’s modified eagle medium (DMEM, Cat # D5796, Sigma-Aldrich) supplemented with 10% foetal bovine serum (FBS, Cat # 10270106, Thermo Fisher Scientific), 1% penicillin/streptomycin (P/S, Cat # P4333, Sigma-Aldrich). Cells were grown to confluency in T175 flasks by incubating at 37°C and 5% CO_2_. BSR-T7 cells were used in both plaque assay and PRNT_80_ methods.

BHK21 cells were cultured in GMEM supplemented with FBS (10%), tryptose phosphate broth (5%; Cat # T8159, Sigma-Aldrich), 2mM L-glutamine (Cat # 25030081, Thermo Fisher Scientific) and P/S (1%), at 37°C with 5% CO_2_. BHK21 cells were employed for NSDV amplification and titration by TCID_50_.

### Serum samples

#### CCHFV-endemic field sera

This study utilized Bulgarian sheep sera (n=1198) collected in a serosurvey in 2017 (14), based on molecular and serological evidence suggesting long-term endemicity of CCHF in the country (25). The study collected samples from two provinces, selected due to reported high levels of CCHFV infection in humans and presence of CCHFV antibodies in livestock (26). Briefly, within each province, 60 farms were selected, and with each farm 5 lambs (<1 year old) and 5 sheep between 1- and 2-years-old (10 total) were sampled for their sera. Tested samples are from a region where CCHFV but neither NSDV nor DUGV is circulating; ensuring suspected cross-reactivity is not instead the result of previous heterologous infections or co-infections. There are no reports of molecular detection in local ticks nor serological evidence from serosurveys that suggests NSDV has previously been, or is currently, present in small ruminant populations in Europe (11). Likewise, reports suggest DUGV to be localized to the African continent (17–20), suggesting antibody responses due to prior NSDV or DUGV infections are highly unlikely. Sheep serum samples were shipped to The Pirbright Institute for serological testing. All samples were heat inactivated at 56°C for 30mins, in accordance with a standard heat-inactivation protocol (27).

#### Control sera from non-endemic area

Sheep sera (n=180) from the United Kingdom (non-endemic) were also included as negative controls. These samples were screened against NP and Gc for both CCHFV and NSDV and cut-offs were estimated.

#### Experimental NSDV-seropositive sera

Sera from an animal study conducted by bin Tarif et al. (24) at The Pirbright Institute were used to assess the potential cross-reactivity of NSDV-specific serum antibodies towards CCHFV antigens. In this study, six sheep were infected with either 10^4^ TCID_50_ NSDV ND66-PC9 (sheep VU15-17) or 10^4^ TCID_50_ NSDV Ganjam IG619 (sheep VU18-20). Three individuals out of six were culled 7 days post-infection (dpi); the remaining three were kept until 11 dpi. Serum samples were collected 0, 7, 9 and 11 dpi. The serum samples taken 0- and 11-dpi from sheep VU18 were used as negative and positive controls respectively when screening Bulgarian sheep antibody responses to NSDV NP and Gc antigens.

### In-house indirect ELISAs

#### Proteic antigen ELISA

The procedure was previously described by Belij-Rammerstorfer et al. (14). Nunc MaxiSorp 96-well plates (Cat # 442404, Thermo Fisher Scientific) were coated with 50µL of the appropriate orthonairovirus or phlebovirus antigen (**Table 1**) at 1µg/mL and left at 4°C overnight. The following day, the antigen solution was flicked off, and 200µL of Blocker™ Casein in PBS (Cat # 37528, Thermo Fisher Scientific) was added to wells; left at room temperature for 2 hours to prevent non-specific antibody binding. After antigen-coated plates were washed four times with PBS-Tween20 0.05% (PBS/T), test sera diluted 1:125 in Blocker™ Casein were added in duplicate. To account for plate-to-plate variation, a blank (no sample, Blocker™ Casein only), NSDV positive samples (VU18 11dpi) and NSDV negative sample (VU18 0dpi) controls were added in duplicate. Once all test sera and appropriate controls were added, plates were then sealed and incubated overnight at 4°C. The next day, sera dilutions were flicked from the test plate, and wells were washed four times in PBS/T. HRP-conjugated Donkey anti-sheep/goat IgG antibody (Cat # STAR88P, Bio-Rad) was employed as a secondary antibody, diluted 1:30 000 in Blocker™ Casein and added to each well at a volume of 50µL. After incubation at room temperature for 1 hour, plates were once again washed four times with PBS/T. TMB substrate (Cat # TMBW-1000-01, Surmodics) was added (50μl/well) and incubated for 10 minutes at room temperature protected from light. Fifty microliters of stop solution (Cat # STPR-1000-01, Surmodics) was added to each well to halt the development of the assay. The optical density was read at a wavelength of 450nm using Gen 5 Synergy™ 2 version 3.10 microplate reader and imager software (BioTek™). OD values obtained were adjusted by subtracting to that obtained for the blank in each plates. Moreover, the ability of CCHFV-specific monoclonal antibodies to react with NSDV Gc was also assessed using the same procedure. Antibodies were obtained from BEI Resources (anti-CCHFV N: clone 2B11 #NR-40257, clone 9D5 #NR-40270; anti-CCHFV Pre-Gn: clone 6B12 #NR-40259, clone 7F5 #NR-40281; anti-CCHFV Gc: clone 30F7 #NR-40288, clone 11E7 #NR-40277, clone 12A9 #NR-40254, clone 13G5 #NR-40293, clone 3E3 #NR-40273, clone 1H6 #NR-40296), and were used at 1:800 dilution.

**Table 1:** *Orthonairovirus* test antigens employed during experiments.

Antigen	Accession (Strain, Country of origin if known)	Expression species	Source, Cat#
NSDV NP	YP_009361831 (Jilin, China)	*Escherichia coli*	Reading University, UK Custom service
NSDV Gn	ACH99800 (708, Kenya)	HEK293 (Human)	The Native Antigen Company, UK #REC31904
NSDV Gc	ACH99800 (708, Kenya)	HEK293 (Human)	The Native Antigen Company, UK #REC31906
CCHFV NP	NP_950237 (IbAr10200, Nigeria)	HEK293 (Human)	The Native Antigen Company, UK #REC31639
CCHFV Gn	AIN41199 (IbAr10200, Nigeria)	HEK293 (Human)	The Native Antigen Company, UK #REC31615
CCHFV Gc	NP_950235 (IbAr10200, Nigeria)	HEK293 (Human)	The Native Antigen Company, UK #REC31696
DUGV NP	NP_690574 (ArD44313, Senegal)	*Escherichia coli*	Cusabio Biotech, #CSB-EP851800DCAH
RVFV NP	YP_003848707 (ZH-548, Egypt)	HEK293 (Human)	The Native Antigen Company, UK #REC31640
RVFV Gn	P21401 (Uniprot) (ZH-548 M12, Egypt)	HEK293 (Human)	Oxford University, UK Gift (29),
RVFV Gc	P21401 (Uniprot) (ZH-548 M12, Egypt)	HEK293 (Human)	Oxford University, UK Gift (29),
Inactivated NSDV	(Entebbe ND66 PC9, Uganda)	BHK21 (Hamster)	Central Veterinary Institute of Wageningen, the Netherlands (24)
Commercial CCHFV Ag	Unknown	Unknown	Vector Best, Russia

#### Inactivated NSDV antigen ELISA

Plates were coated overnight at 4°C with 100μl of PFA solution (final concentration 2% in PBS) containing NSDV (at an equivalent of 0.5 ×10^5^ TCID_50_). The day after, coating solution was removed from the plates, washed 3-4 times with PBS/T, blocked using PBS/T containing 4% skim milk (PBS/T-milk) for at least one hour. Serum samples were diluted at 1:800 in PBS/T-milk, added onto plates, one well per sample, after removing blocking solution. The experiment was performed three times. Sera were incubated for an hour in the plates, then washed 3-4 times. A HRP-conjugated Donkey Anti-Sheep IgG (whole molecule)–Peroxidase antibody (Sigma, Cat. No. 3415) was diluted at 1:10 000, added into the wells and incubated for an hour. Plates were washed then developed using TMB and stop solution as above.

#### VectoCrimean-CHF ELISA

The sera were tested using VectoCrimean-CHF IgG (Vector Best) following a procedure adapted as in Mertens et al. (28). Briefly, after the incubation time with the sera and the washes, the HRP-conjugated Donkey anti-sheep/goat IgG antibody (Cat # STAR88P, Bio-Rad) was used at a dilution of 1:5000. Then plates were washed and developed using TMB and a stop solution.

### NSDV plaque assay

BSR-T7 cells were seeded at 3×10^5^/well in a 24-well tissue culture plate (Cat # 353047, Scientific Laboratory) and incubated overnight at 37°C, 5% CO_2_. Subsequently, culture DMEM was removed, cells were washed once with PBS, before 200µL of 2-fold serial diluted NSDV ND66 PC9 were added to corresponding wells and incubated for one hour. Once viral dilutions had been removed, each well was washed with PBS, sealed with 1% agar diluted 1:4 in culture DMEM and left to incubate for 4 days. Agar plugs were detached, and crystal violet solution was added to each well for 30 minutes. Crystal violet was removed, and wells were washed with sterile water. Plates were scanned and plaques were counted manually. A stock titre of 3.79×10^5^ or 10^5.6^ plaque forming unit per mL (PFU/mL) was calculated as an average over three technical repeats for NSDV ND66 PC9.

### PRNT_80_ NSDV

BSR-T7 were seeded at 3×10^5^ cells/well in 24-well plates (Cat # 353047, Scientific Laboratory), alongside sera samples being heat-inactivated for 1 hour at 56°C the day before testing. Two-fold serial dilutions of sheep sera were prepared in incomplete FBS-free DMEM ranging from 1:8 to 1:64. A virus solution of NSDV ND66 was prepared at 1:2560 dilution from the stock, then a volume containing ~19 PFU was gently mixed to an equal volume of diluted serum. This quantity of virus was determined through protocol optimization and is the resultant of the dilution at which the amount of virus is sufficient to allow quantification of a 80% reduction without inducing a complete lysis of the cell monolayer. Serum/virus mix was left to incubate at room temperature for 20 minutes before being added onto cells at 200 μl per well and incubated at 37°C 5% CO_2_ for an hour. Each plate contained a duplicate positive infected controls (no serum) and negative non-infected controls (no virus, no serum). The subsequent revelation procedure was that of the plaque assay described above. For a sample to be classified positive, there must be a reduction of ≥ 80% in PFU compared to the positive control (PRNT_80_). Because there was no information on an expected proportion of positives by PRNT_80_ within the CCHF-endemic sheep serum field sample set, we have randomly screened 224 samples with a O.D. value > 0.2 on NSDV Gc-specific ELISA; that would allow us to pick up a proportion as low as 18% ± 5% (margin of error) with a 95% confidence.

### Epitope collection and similarity plot

CCHFV NP- and Gc-derived antibody epitopes were searched through articles on PubMed (see **Supplementary Table S2**). The words “CCHF epitope” and “CCHFV epitope” were used, returning 28 articles dealing with epitopes (search results as of January 2024). Out of 28 articles, 15 present experimentally identified epitopes while the remaining 13 articles report on predicted epitopes. Overall, only 4 articles report testing antibody epitopes bound by sheep sera and were subsequently used for plotting the location of epitopes recognized by sheep antibodies. Similarity plots represent the percentage of identity in a moving window of 20 amino acids with a step size of 1, covering an entire alignment of NP & Gc from CCHFV IbAr10200 strain (Genbank Accession Number, NP: NP_950237; Gc: NP_950235.1) and NSDV Jilin and 708 strains (Genbank Accession Number, NP: YP_009361831; Gc: ACH99800, respectively). The alignment was performed at the amino acid level using the ClustalW algorithm in the software MEGA 7.0 and was exported in a tabular file. The positions of epitopes targeted by sheep serum and human monoclonal antibodies from the literature are indicated (see Amino acid alignments in **Supplementary Material**).

### Statistical test

To assess correlation between anti-CCHFV and anti-NSDV in-house ELISAs, OD values for both NP- and Gc-specific in-house ELISAs were compared independently using the Spearman’s rank test. Jarque-Bera test was used for testing normality of the distribution of OD values of serum responses to CCHFV NP, CCHFV Gc, NSDV NP and NSDV Gc. The version of R employed was R.4.2.0 (R Core Team, 2022). Spearman’s rank correlation coefficient (Rho) was computed using R. Graphical representation was performed using Jupyter Notebook 7.0.8 and Python 3.12. In addition, Kappa statistic was used to assess correlation between anti-CCHFV and anti-NSDV in house ELISAs. Classification (positive/negative) for both NP and Gc-specific ELISAs were compared independently, and field samples, negative control samples and experimental samples were assessed separately. Analysis was conducted in R using package epiR (function epi.kappa). To assess the repeatability between plates and between wells technical repeats, inter-assay coefficient of variation (CV), and intra-assay CV values were calculated. These are provided as supplementary material (**Supplementary Table S3**).

## Results

### Cross-reaction among CCHFV-endemic field sera

**Figure 1:**
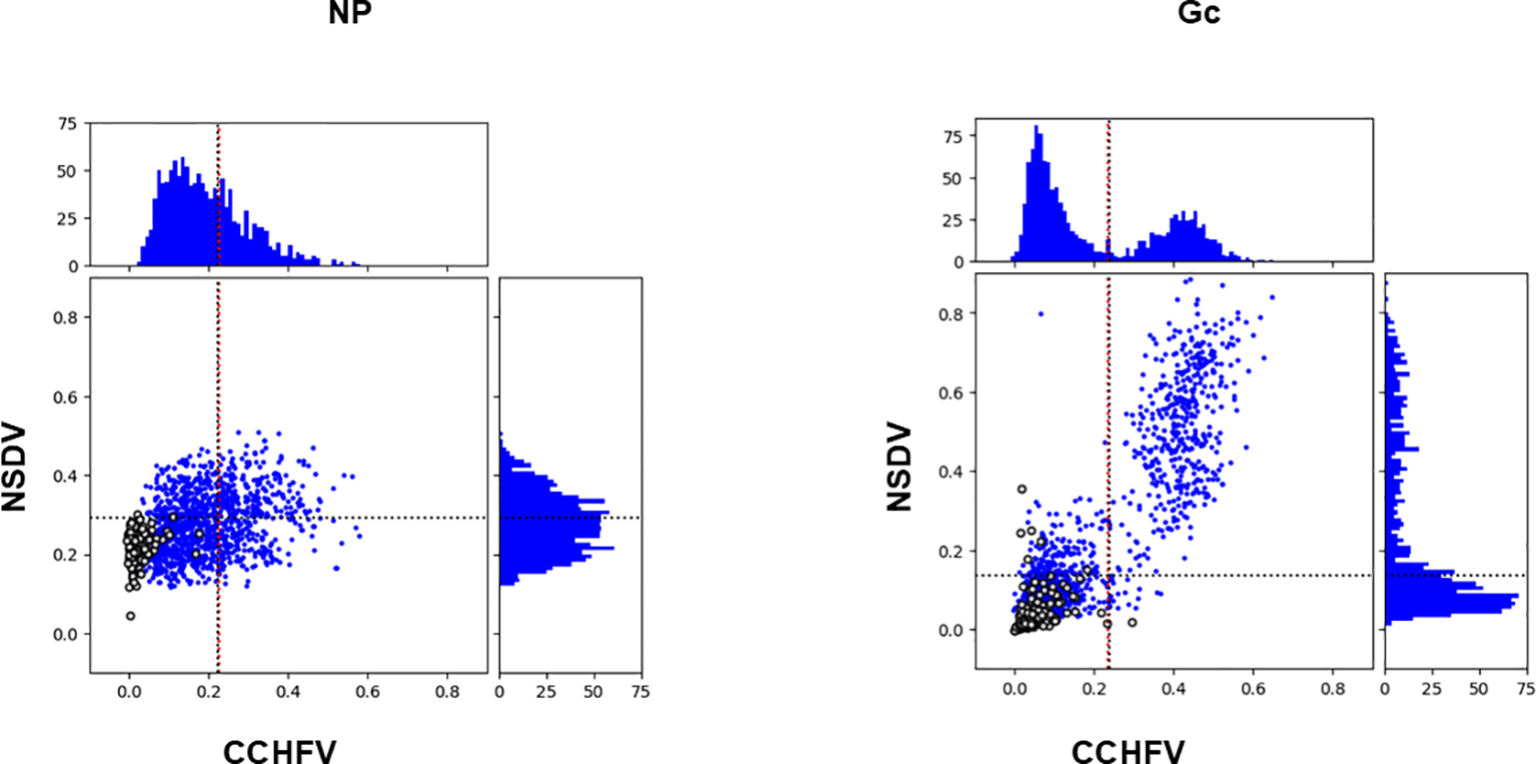
Antibody responses to NSDV and CCHFV NP (left) and Gc (right) among CCHF-endemic field sera (n=1198). For each antigen, the top and right quadrant represent the frequency distribution of antibody responses based on their OD values towards CCHFV and NSDV, respectively. The quadrant in the middle represent each serum OD value from NSDV-specific ELISA plotted against its respective OD value from CCHFV-specific ELISA. Sera from CCHFV endemic area are represented as blue dots while sera from non-endemic area are represented as open circles. All sera were used at a 1:125 dilution.

CCHFV-endemic field sera (n=1198) previously tested against CCHFV NP and Gc (14) were assayed against NSDV NP and Gc (this study; **Figure 1**). The distributions of OD value for all four antigens were not normally distributed (Jarque-Bera test, p < 0.05). Anti-CCHFV NP-specific and anti-NSDV Gc-specific were skewed to the right while CCHF-Gc specific exhibit a bimodal distribution (**Figure 1**). Hence, Spearman’s correlation test was performed to assess whether a correlation exists between OD values from both assays, i.e. whether sera strongly reacting to CCHFV NP and Gc tend to strongly react to respective NSDV antigens. A strong Spearman’s correlation coefficient was obtained when CCHFV Gc values were tested against NSDV Gc (Rho = 0.810, p < 0.001). Moreover, two populations of animals whose sera were reactive to both NSDV and CCHFV Gc could be observed (**Figure 1**, right panel). Conversely, a smaller correlation coefficient was found when testing CCHFV NP against NSDV NP values (Rho = 0.270, p < 0.001). Notably, only a diffuse population could be observed with a shift towards high CCHFV OD values but not necessarily high NSDV OD values.

In our previous study (14), a cut-off value for positivity was estimated using a finite mixture model for both CCHFV NP (0.234) or Gc (0.225). Such cut-off values were similar to the average OD value plus two standard deviation (μ + 2δ) obtained for sheep sera taken from sheep in the UK where CCHFV is absent (for NP: 0.239; for Gc: 0.222). We reported 413 and 461 positives samples out of 1200 sera for CCHFV NP and Gc, respectively. Based on this observation, we have included a set of sheep sera from the UK (n = 180), that have been screened against NP and Gc for both NSDV and CCHFV (**Figure 1**, open circles). We have estimated a cut-off value for NSDV NP and Gc using the μ + 2δ formula applied to the 180 UK sheep sera herein. Cut-off values were 0.293 for NSDV NP and 0.139 for NSDV Gc. We then applied this cut-offs to estimate the amount of sera that classify as positive for NSDV NP and Gc within the 410 and 460 sera that were classified as positives for CCHFV NP and Gc, respectively. 235 sera were positive for NSDV NP out of 410 CCHFV NP positives (57.3%), while 439 sera were positive for NSDV Gc out of 460 CCHFV Gc positives (95.4%).

Furthermore, to quantify the agreement between the assays (as a proxy for cross-reaction), Cohen’s Kappa test was employed following classification of positive/negative, to the CCHFV-endemic field sera (n=1198) and sheep sera from the UK (n = 180). Regarding CCHFV-endemic field samples, the test reported a fair agreement between CCHFV NP and NSDV NP assays (Kappa statistics = 0.209), while it reported a substantial agreement between CCHFV Gc and NSDV Gc (Kappa statistics = 0.681) (see **Supplementary Table S4**). Using the sheep sera from the UK, only one sample was classified as positive for CCHFV Gc and six for NSDV Gc, while none was positive for CCHFV NP and two for NSDV NP (**Supplementary Material Table S4**).

### Experimentally NSDV-infected sheep sera cross-react with CCHFV Gc

**Figure 2:**
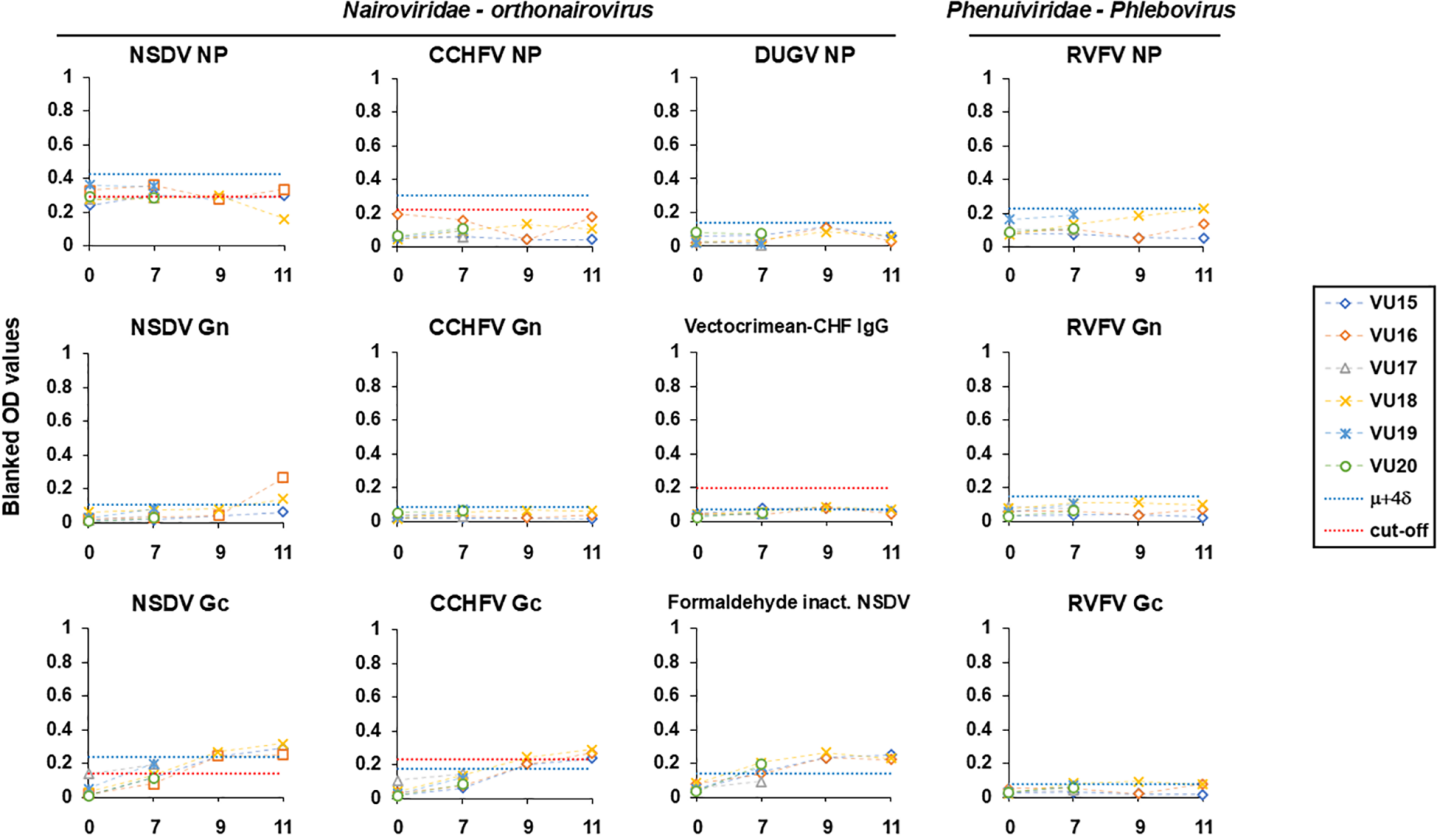
Antibody responses following NSDV infection (0, 7, 9, 11 dpi) towards antigens of NSDV (NP, Gn, Gc, inactivated virus), CCHFV (NP, Gn, Gc, CCHFV commercial antigen), DUGV (NP) and RVFV (NP, Gn, Gc). Each individual response is represented as blanked OD values. The blue dashed line indicates the a set cut-off equal to the average OD value taken at 0 dpi (before infection) plus four standard deviation (μ + 4δ). The red dashed line indicates the cut-off instructed in the kit (VectoCrimean-CHF IgG), previously established for CCHFV NP and Gc-specific ELISAs (14), or as estimated using n=180 UK negative sheep sera (this study). A dilution of 1:125 per serum was used for all ELISAs except for formaldehyde-inactivated virus-based ELISA where 1:800 was used.

To get an insight into the cross-reactive potential of NP-specific and Gc-specific, we tested samples collected from British sheep infected by NSDV in a control experiment setup (24) against several antigens (**Figure 2**, also see **Supplementary Figure S1** and **Supplementary Table S1** for values). The list included orthonairovirus antigens represented by NSDV (NP, Gn, Gc) and formaldehyde-inactivated NSDV; CCHFV (NP, Gn, Gc) and a CCHF commercial antigen; DUGV NP; and antigens of a distant bunyavirus modelled by RVFV (NP, Gn and Gc). We have set a cut-off value for all antigens that correspond to the average OD value taken at 0 dpi (before infection) plus four standard deviation (μ + 4δ) to be stringent. We have also included cut-offs that are established already for CCHFV commercial ELISA, CCHFV NP and Gc (14); or have been estimated using a set of UK negative sheep sera (n=180) for NSDV NP and Gc (this study). All OD values for responses to all NPs did not increase for the entire course of infection and remained below the set cut-off. For NSDV NP, most responses were above the estimated cut-off using UK negative sheep sera from 0 dpi. This suggestive of an absence of responses mounted in comparison to day 0. Regarding responses to Gn, antibody response from two animals towards NSDV Gn spiked at 11 dpi and were above the set cut-off (VU16 and VU18). The responses towards CCHFV and RVFV Gn remained below the set cut-off. When testing against Gc, sheep responses towards NSDV and CCHFV Gc were very similar, displaying a constant rise across the course of infection for all sheep individuals that survived up to 11 dpi (VU15; VU16 and VU18), reaching the set cut-off by 7 dpi and remaining above both set and established cut-offs at 11 dpi. This is suggestive of a seroconversion in all this animal. This observation was confirmed against inactivated NSDV. VectoCrimean-CHF IgG assay displayed antibody responses that remained weak at all times and below both set and the instructed cut-off from manufacturer’s instruction at 11dpi. As observed for Gn, responses to RVFV Gc remained weak despite two animals displaying OD values by the set cut-off at 11 dpi. To get more insight into these antibody responses, we have also analyzed the response of each animal during the course of infection as a fold-increase to the response measured at 0 dpi. The responses observed at 11 dpi were substantially higher than that at 0 dpi for NSDV Gc, NSDV Gn (2/3 animals) and CCHFV Gc, displaying more than a 5-fold increase (**Supplementary Figure S1**). Altogether, for all sheep sera available by 11 dpi, similar responses were measured towards NSDV Gc and CCHFV Gc. Since these sheep were only exposed to NSDV, our observation is suggestive of a particular cross-reaction of NSDV-seropositive individual towards CCHFV Gc. Moreover, while antibodies to NP are not yet detectable by 11dpi, as previously suggested (22), antibodies to Gc are detectable, depicting potential differences in response dynamics to different antigens.

### CCHFV-seropositive sera do not cross-neutralize NSDV

**Figure 3:**
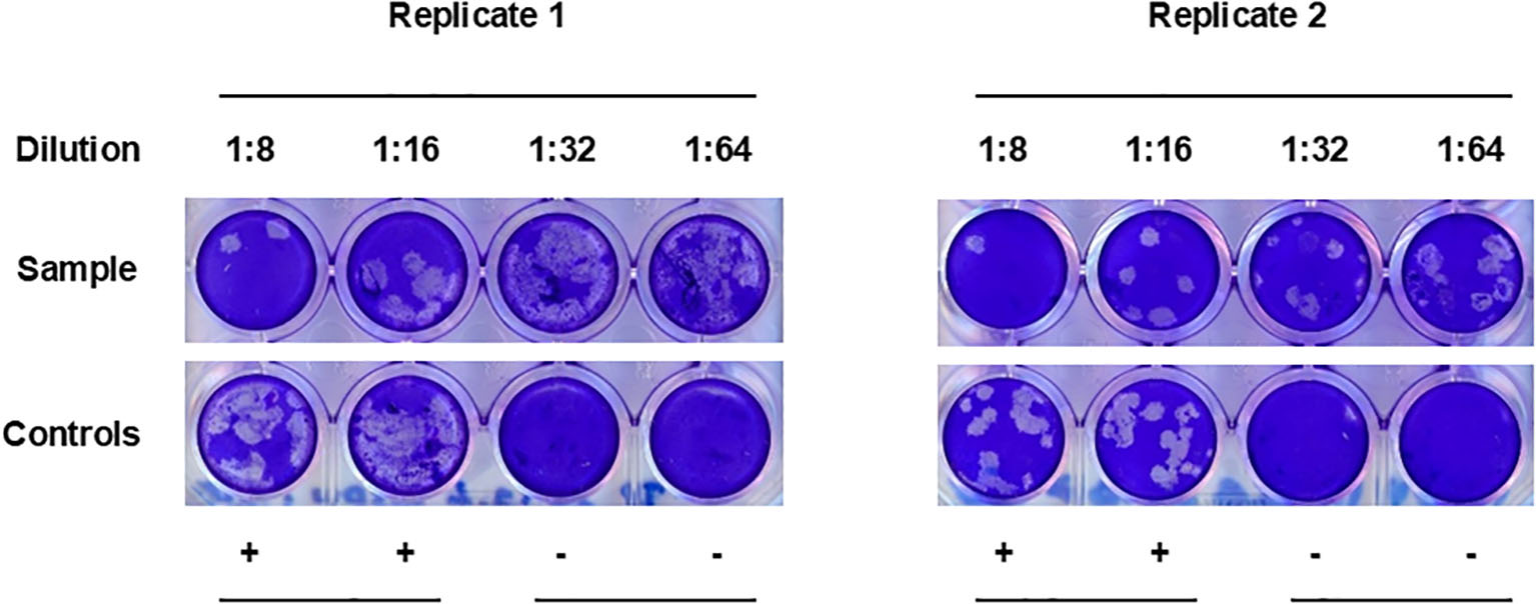
PRNT_80_ cross-neutralization of NSDV *in vitro*. Serum samples were 2-fold diluted from 1:8 to 1:64. Each dilution was tested in single wells and the test was performed twice independently. In each plate, duplicate wells for positive infected controls (“+”; no serum) and negative non-infected controls (“-“; no virus, no serum) were included. The figure represents the only serum able to cross-neutralize NSDV in the selected group of samples tested (n=224).

It has been suggested that antibodies able to neutralize CCHFV infection *in vitro* are mainly directed towards epitopes in the Gc (30). Therefore, we wanted to test whether Gc ELISA cross-reactive sheep sera from CCHFV-endemic areas were able to cross-neutralize NSDV *in vitro*. To achieve this goal, we randomly selected a cohort of 224 sera that displayed high OD values (> 0.2) to NSDV Gc, and subsequently test their ability to neutralize NSDV using an in-house PRNT_80_ assay. Only one serum out of the 224 tested (0.4%) demonstrated a neutralizing capacity at the lowest dilution of 1:8 but not above (**Figure 3**). This suggested that sheep possessing serum antibodies against CCHFV Gc that cross-react to NSDV Gc, are not able to neutralize an NSDV infection, or only marginally.

### Analysis of published sequences of antibody epitopes in the immunodominant region of NP reveals they are highly dissimilar between CCHFV and NSDV

**Figure 4:**
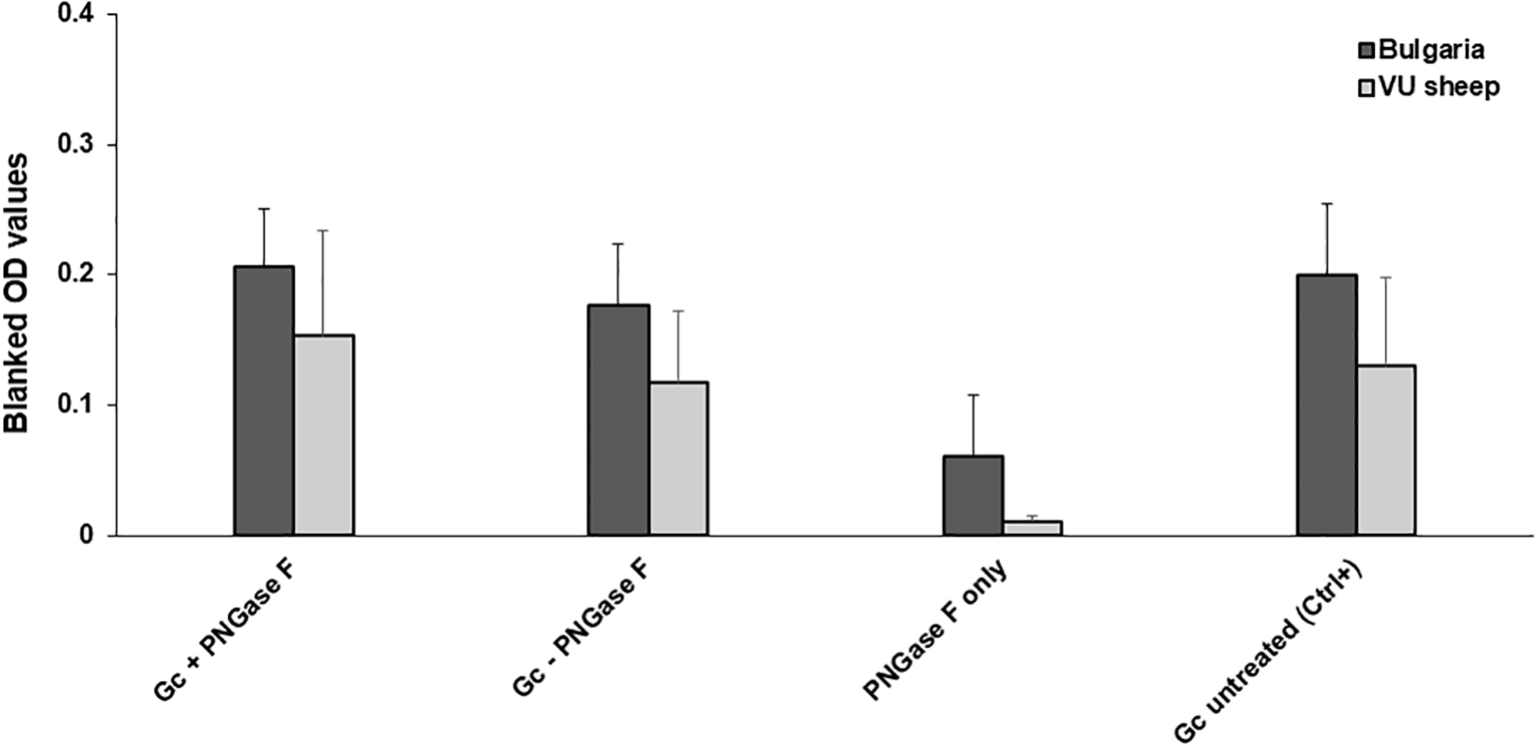
PNGase F treatment does not affect Gc cross-reaction. NSDV Gc was incubated at 37°C in the presence or absence of PNGase F, then used to coat ELISA plates. Reactivity of n=10 Bulgarian cross-reactive sera and n=3 sera from NSDV-infected sheep (11dpi) was assessed. Reactivity to the enzyme alone and untreated protein were included as controls.

**Figure 5:**
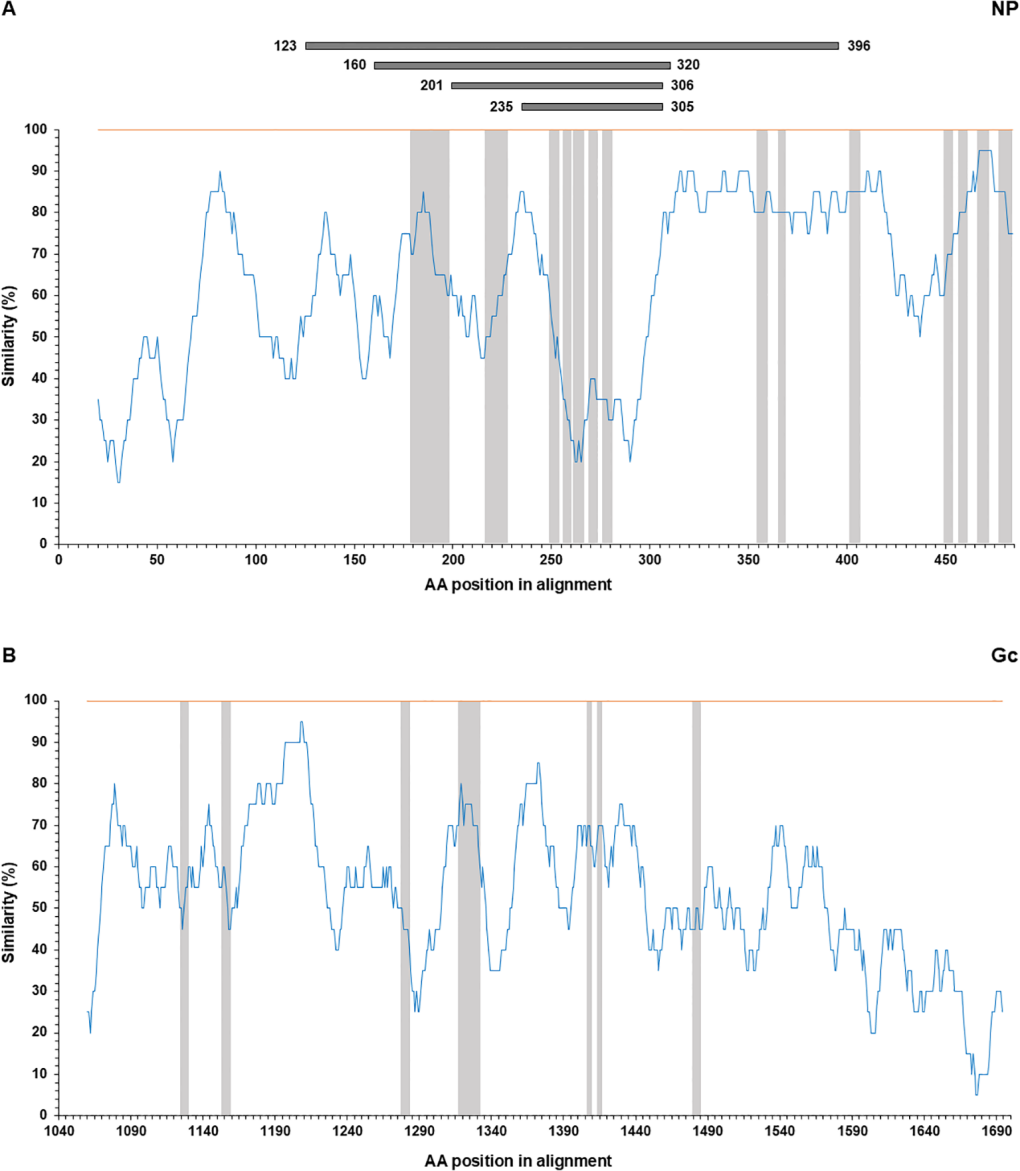
Similarity plot between CCHFV (orange) and NSDV (blue). Top panel **(A)** NP and bottom panel **(B)** Gc, using the percentage of identity in a moving window of 20 amino acids with a step size of 1. Epitope locations reported for antibodies from sheep serum are highlighted in gray. For NP, immunodominant regions reported from four separate studies (31–34; **Table 2**) are indicated on top of the plot in grey.

**Figure 6:**
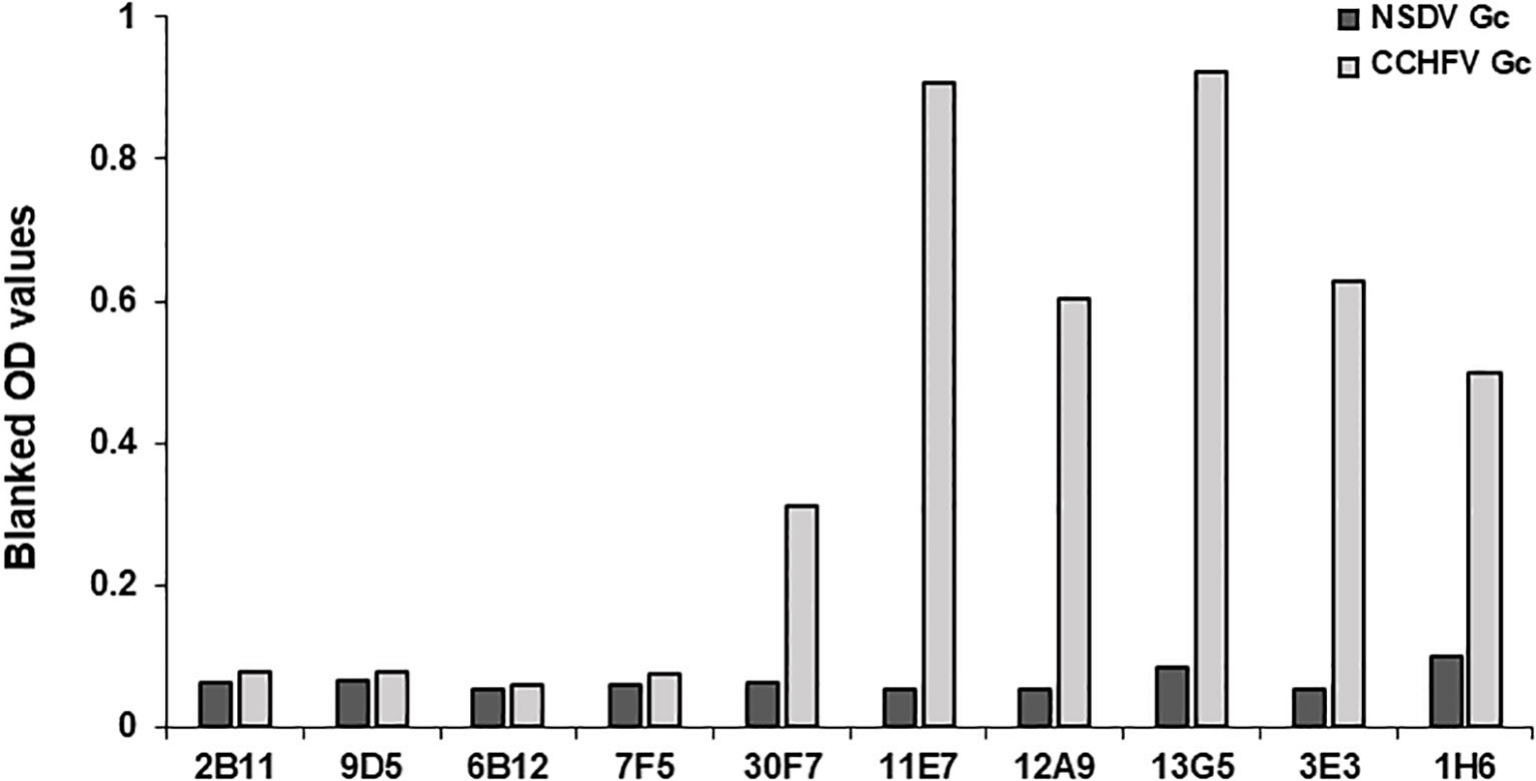
anti-CCHFV Gc mAbs binding conformational epitopes do not cross-react to NSDV Gc. Gc from NSDV or CCHFV were coated onto ELISA plates and reactivity of mouse-derived CCHFV-specific mAbs was assessed at a dilution of 1:800. MAbs were obtained from BEI resources and are as follows: 2B11, 9D5 are CCHFV NP-specific, 6B12, 7F5 are Pre-Gn-specific (38) and 30F7, 11E7, 12A9, 13G5, 3E3, 1H6 are CCHFV Gc-specific. MAbs used were obtained from BEI resources. All mAbs were used at a 1:800 dilution.

The nucleoprotein is more conserved among orthonairoviruses than the Gc (60% and 52.3% respectively, see alignment provided in **Supplementary**), however our data presented so far suggests a lesser cross-reaction to the NP than the Gc. In addition, Gc is glycosylated while NP is not. As such, we investigated the distinctive N-linked glycosylation of the Gc antigen as a reason for a higher recorded cross-reactivity compared to the NP antigen. **Figure 4** shows that the treatment of the recombinant NSDV Gc by PNGase F did not abrogate reactivity of either cross-reactive sheep sera from CCHFV-endemic field area, nor NSDV seropositive sera, suggesting glycans harboured by Gc are not responsible for its cross-reactive potential. Another conceivable hypothesis explored was that sheep mount an antibody response against NP epitopes that differs between orthonairoviruses while antibodies directed towards epitopes in Gc are mainly conserved. To get an insight into this, publications that studied anti-CCHFV NP and Gc antibody targets were identified from PubMed database. Fifteen articles determined regions and precise epitopes bound by antibodies from different species (mouse, human, sheep, goat, cattle – **Supplementary Table S2**). Only four studies mapped linear epitopes bound by serum from sheep with a history of CCHFV exposure (see **Supplementary Table S2**, green highlights). For NP, it has been suggested that the central portion of the protein is immunodominant (31–34; **Table 2**), and so the majority of antibodies are directed against that region. In this region, most epitopes identified using sheep sera present highly divergent motif sequences to CCHFV (**Figure 5A**). In addition, there are epitopes near the C-terminus, in a region of high similarity outside the immunodominant region of NP; these are potentially involved in the weak cross-reactivity displayed by NP. Regarding the Gc, there was only one study that assessed sheep serum antibody binding epitopes (**Supplementary Table S2**). Of the 7 reported epitopes, only 2 were found in a portion of Gc that are relatively conserved (>70%; **Figure 5B**; **Supplementary Table S2**). However, we could not find any study reporting the location of the immunodominant region(s) of Gc. Furthermore, all epitopes targeted by sheep antibodies that have been reported are linear. Two articles detail conformational epitopes in the Gc using human-derived monoclonal antibodies (30, 35). When aligning amino acid sequences of CCHFV and NSDV Gc used in our study, half of the binding motifs of these monoclonal antibodies were identical (10/20 and 9/17, respectively). However, when testing mouse monoclonal antibodies known to bind conformational epitopes (36), they did not react to NSDV Gc (**Figure 6**). Altogether, presented data suggest that the NP is lowly cross-reactive due to dominant epitopes being highly dissimilar between CCHFV and NSDV. However, Gc cross-reactivity requires further investigation.

**Table 2:** Immunodominant portion of CCHFV NP.

Position on CCHFV NP (aa)	Species-derived serum used	Reference
235-305	Rabbit (immunization)	(31)
201-306	Human (CCHF patients)	(32)
160-320	Human (CCHF patients)	(33)
123-396	Human (CCHF patients)	(34)

## Discussion

Small ruminants act as hosts and ‘reservoirs’ for several *Orthonairovirus* genus member viruses and as such, are often the target of surveillance programmes which utilize serological assays to indicate the presence of circulating antibodies. Cross-reactivity between members of the *Orthonairovirus* genus has been demonstrated to reduce the specificity of virus-specific serological assays, both experimentally (22) and in the field (in regions where more than one orthonairoviruses are circulating), leaving unclear if what is observed is cross-reaction or co-infection (23). To advance such work, we focused on serum antibody responses to CCHFV and NSDV, employing field sheep sera from a region where CCHFV circulates (25) but NSDV is evidently absent (11), eliminating the potential effect of co-infection when studying cross-reactivity. To assess the cross-reactivity of NP and Gc antigens, in-house NP-specific and Gc-specific ELISAs as well as a PRNT_80_ were developed for NSDV. First, a large subset of sheep field sera sampled from a region where CCHFV is endemic and NSDV is known to be absent was utilized; allowing the quantification of the effect cross-reactivity is having on these assays, by comparing previously tested anti-CCHFV responses (14) with anti-NSDV responses.

A significant positive correlation between anti-CCHFV and anti-NSDV Gc-specific responses of CCHFV-endemic field sera tested on our in-house Gc-specific ELISAs indicated a strong cross-reactivity of antibodies towards Gc. To confirm this observed cross-reaction in field sera, a second subset of sera that have been harvested during the course of a controlled NSDV inoculation in sheep, were tested against both NSDV and CCHFV Gc antigens on in-house ELISAs. Our results show NSDV infected sheep display a seroconversion of anti-NSDV Gc-specific antibodies across 11 days. Meanwhile and despite a low sample size, the presence of NSDV-specific antibodies able to react to the CCHFV Gc supported evidence of this cross-reaction that could be seen in field samples. Furthermore, Cohen’s Kappa test reported a substantial agreement (Kappa statistics = 0.769) for CCHFV Gc and NSDV Gc assays performed on these experimental samples (**Supplementary Table S4**). These results validate previous evidence of orthonairovirus glycoprotein cross-reactivity, with DUGV seropositive samples previously testing positive on a commercial species-adapted immunofluorescence assay based on CCHFV glycoprotein-expressing cells (23). Data presented in this study also demonstrates Gc-specific ELISAs to have a reduced specificity when tested against NSDV in a population where only anti-CCHFV responses are expected. In regions where two or more orthonairoviruses overlap in their distribution ranges, such findings suggest Gc-based ELISAs would be potentially ineffective in correctly identifying which *Orthonairovirus* genus member is circulating in small ruminant populations. Therefore, confirmation using a virus neutralization test such as PRNT_80_ would be highly recommended in these settings. Despite being subject to cross-reactions, Gc-based ELISA remains a valuable tool for studying the nature of immune responses in domestic animals, notably when the nature of viruses circulating in the area is known or in a controlled experimental setup.

To assess whether N-linked Glycosylation of the Gc was responsible for cross-reactivity observed in our previous experiments, a small subset of ‘high’ anti-NSDV responding sera were tested against Gc with its N-linked glycosylation removed. When compared to the naïve Gc control, no significant difference in OD value was observed. These results suggested that this glycosylation was not the cause of exhibited cross-reactivity and instead, a conserved epitope is responsible for such a cross-reaction with further research necessary to identify this. Nonetheless, it appears that NP is less cross-reactive due to its dominant epitopes being highly dissimilar between CCHFV and NSDV.

NP-based assays, in particular NP-based ELISAs, have previously been demonstrated to have high specificity for individual orthonairoviruses when tested against highly related species experimentally (22) and in the field (23). However, to confidently estimate the effect such cross-reactivity is having in a field setting, without the effect of co-infection on our sample set, our large CCHFV-endemic field sera sample set was used to assess the CCHFV/NSDV cross-reaction model. A weakly positive correlation between anti-CCHFV and anti-NSDV NP-specific responses of CCHFV-endemic field sera was observed on our in-house NP-specific ELISAs. To confirm this evidence of a weak cross-reaction between NSDV and CCHFV NP, sera from sheep experimentally infected against NSDV were tested against CCHFV and NSDV NP on in-house ELISAs. No seroconversion of NSDV NP antibodies occurred over 11dpi and so as expected, no CCHFV NP responses were also identified. The lack of seroconversion of NSDV NP antibodies prior to 11dpi followed evidence from Hartlaub et al. (37). The authors suggested a seroconversion of anti-NSDV NP-specific antibodies further into the NSDV infection timeline, with antibodies of high-dose inoculated sheep first detectable in all sheep 15dpi. Data presented here continues to support NP-based ELISAs as the most specific test antigen for field ELISAs in comparison to Gc-based ELISAs. However, there are indications that the dynamics (Gc-specific responses appeared earlier than NP, this study) and nature of antibody responses (there are proportions of animals that are seropositive for Gc but not NP, see 14) may differ based on the animals and antigen screened. Future experiments must be conducted over a longer infection period to effectively assess cross-reactions over the NP seroconversion timeline.

DUGV and Hazara virus (HAZV) anti-sera have previously been reported to cross-neutralize NSDV infection, suggesting anti-orthonairovirus responses to be capable of cross-neutralizing infection of highly related *Orthonairovirus* genus members (22). To see whether CCHFV anti-sera was capable of neutralizing NSDV *in vitro*, a subset of ‘high’ anti-CCHFV and anti-NSDV responding sera on our in-house NP- and Gc- based ELISAs were tested on an in-house PRNT_80_ assay. Only marginal cross-neutralization of NSDV was observed, with only 1/224 CCHFV-endemic field samples testing positive on this test format. Neutralization tests are time consuming, require trained laboratory personnel and must be carried out in appropriate containment conditions (BSL2: NSDV, BSL4: CCHFV). In addition, previous reports of strong cross-neutralization between NSDV and highly related co-circulating *Orthonairovirus* genus members such as DUGV (22) restricts this assay for use in regions where only NSDV is present in small ruminant populations.

## Conclusion

In this report, we assessed the existence of a cross-reactivity of sheep serum antibodies towards CCHFV and NSDV antigens as well as assess cross-neutralization of CCHFV-endemic field sera against NSDV using an in-house neutralization test. While the Gc exhibited a strong cross-reaction, the NP was weakly cross-reactive, hence being the antigen recommended for serological assays to distinguish between CCHFV and NSDV in regions where both co-circulate. The lack of cross-neutralization using our in-house PRNT_80_ suggested positive samples were indeed cross-reactive and not co-infected. Due to limitations with required laboratory conditions, we suggest this assay could be used as a confirmatory test following initial screening using ELISA tests in areas where many *Orthonairovirus* are circulating. Finally, while few studies have unravelled the amino acid sequence of the immunodominant region of NP targeted by serum antibodies, the same should be carried out for Gc, potentially revealing epitopes responsible for this cross-reaction. This may allow the design of a recombinant antigen capable of mitigating this phenomenon.

## Data Availability

The raw data supporting the conclusions of this article will be made available by the authors, without undue reservation.

## Supplementary material

Supplementary Figure 1 Fold-increase of sheep antibody responses to various bunyavirus antigens following NSDV infection.

Supplementary Table 1 Blanked OD values for each viral antigen.

Supplementary Table 2 Conservation of epitopes.

Supplementary Table 3 Inter-assay and intra-assay coefficient of variation Amino acid alignments: Amino acid alignment of CCHFV and NSDV NP and Gc.
